# Demographic, tumour, and treatment characteristics of female patients with breast cancer in Sri Lanka; results from a hospital-based cancer registry

**DOI:** 10.1186/s12885-021-08929-8

**Published:** 2021-11-03

**Authors:** Don Thiwanka Wijeratne, Sanjeeva Gunasekera, Christopher M. Booth, Hasitha Promod, Matthew Jalink, Umesh Jayarajah, Sanjeewa Seneviratne

**Affiliations:** 1 Department of Medicine, Queen’s University, Kingston, Canada; 2 National Cancer Institute, Maharagama, Sri Lanka; 3 Department of Oncology, Queen’s University, Kingston, Canada; 4 Division of Cancer Care and Epidemiology, Queen’s University Cancer Research Institute, Kingston, Canada; 5 Health Information Unit, Ministry of Health, Colombo, Sri Lanka; 6 Department of Surgery, Faculty of Medicine, University of Colombo, Kynsey Road, Colombo 08, Sri Lanka

**Keywords:** Breast cancer, Cancer registry, Electronic Database

## Abstract

**Background:**

Although breast cancer is the most common cancer among Sri Lankan women, there is little published data on patient characteristics and treatment in the local context. We aimed to describe disease characteristics and management in a large contemporary cohort of women with breast cancer at the National Cancer Institute of Sri Lanka (NCISL).

**Methods:**

All women with invasive primary breast cancers diagnosed during 2016–2020 were identified from the NCISL breast cancer registry. The NCISL sees approximately 40% of all cancer patients in Sri Lanka. Cancer stage at diagnosis was defined according to the Tumour, Node, and Metastasis (TNM) staging system and the Estrogen (ER) and progesterone (PR) receptor status was determined based on the results of immunohistochemistry tests. Descriptive statistics were used to describe the study cohort and treatment patterns.

**Results:**

Over 5100 patients were diagnosed with breast cancer during the study period at the NCISL. The mean age of the women was 56 (SD 12) years. Common co-morbidities were hypertension (*n* = 1566, 30%) and diabetes mellitus (*n* = 1196, 23%). Two thirds (66%) of the cancers were early stage (stage I & II) at diagnosis. ER/PR positivity rate was 72% and HER-2 positivity rate was 22%. Two thirds of the women had undergone mastectomy while 68% had undergone axillary clearance. The rate of chemotherapy delivery was 91% for women with node positive disease while 77% of eligible women (i.e., after wide local excision or with > 3 positive lymph nodes) had received adjuvant radiotherapy. Endocrine therapy was initiated in 88% of eligible women with hormone receptor positive disease while rate of trastuzumab use was 59% among women with HER2 positive breast cancer.

**Conclusions:**

High percentage of advanced breast cancer at diagnosis and high prevalence of comorbidities are some of the major challenges faced in the management of breast cancer in Sri Lanka. Given that stage at diagnosis is the most important prognostic factor determining survival, greater efforts are needed to promote early diagnosis of breast cancer. Considerable lapses in the concordance between guideline recommendations and the delivery of cancer care warrants closer assessment and intervention.

## Introduction

Breast cancer is the most common cancer among Sri Lankan females with an incidence of 24.7 per 100,000 age-standardized population in 2010 [1]. Analysis of the Sri Lanka National Cancer Registry has shown that breast cancer incidence is rising with over 3000 new cases being diagnosed annually [1]. Compared with more developed countries, breast cancer survival rates are relatively low in Sri Lanka, possibly due to multiple factors including lack of availability and delays in diagnosis and treatment [2].

Sri Lanka has primarily a publicly funded healthcare system which provides universal free healthcare for all citizens. Although a private health system is available in the country, the vast majority of cancer cases are diagnosed and managed in the public system; leading to a considerable system-level burden [3]. Cancer care pathways and services have expanded both in the public and private sectors over last two to three decades with substantial improvements both in types of treatment and numbers of patients treated [3].

Recent changes in the incidence of breast cancer and mortality have been observed globally, which somewhat differs between developed and developing countries. The overall incidence of breast cancer has been increasing globally which is largely due to a rapid increase in incidence in developing countries whereas in developed countries incidence rates have plateaued or declining [4]. Similarly mortality rates have seen a major decline in developed countries due to early diagnosis and advancements in therapy, while mortality rates are rising steadily in developing countries including the South Asian region [5, 6]. Population based mammographic screening programmes have contributed towards earlier diagnoses and possibly towards higher incidences in some countries due to over-diagnosis [5]. Although there is some controversy, population-based screening programmes in developed countries appear to have caused a significant reduction in mortality due to early detection of breast cancer [5]. However, this has led to a considerable increase in the incidence of breast cancer due to the increased diagnosis of subclinical disease. Many developing countries including Sri Lanka do not have a nationwide screening program for breast cancer. Although clinical breast examination is available for Sri Lankan women in community-based ‘well women clinics’, utilisation remains low [3]. This may have led to a comparatively higher proportion of breast cancers being diagnosed at an advanced stage when compared to developed countries [7].

Data on the disease characteristics and treatment patterns of breast cancer are scarce in Sri Lanka. Few previous studies have assessed the histological patterns, but have found a higher proportion of triple-negative breast cancers (30–40%) and higher grade neoplasms among Sri Lankan women with breast cancer, which is comparable with other developing Asian countries [8–10]. These prior studies were limited by small sample size and older historic data. Studies on patterns of treatment for breast cancer including conformity to guidelines are nearly non-existent. Data on disease characteristics and treatment patterns are essential to develop effective strategies improve quality of care and outcomes from breast cancer. We undertook the current study to describe the disease characteristics and treatment patterns in a large contemporary cohort of women with breast cancer in Sri Lanka. We also sought to understand treatment patterns in order to evaluate concordance with standard guidelines recommendations.

## Methods

### Study population

All women with newly diagnosed invasive primary breast cancers from 01/01/2016 to 31/12/2020 were identified from the National Cancer Institute of Sri Lanka (NCISL) breast cancer registry. The NCISL breast cancer registry is a prospectively maintained database of an inception cohort that includes all breast cancers treated at the NCISL from 2016 onwards. The NCISL is the largest dedicated cancer hospital in Sri Lanka which provides treatment to approximately 40% of all cancer patients in Sri Lanka. Details on establishing the database, data capture and validation is published elsewhere [11]. Comprehensive information on patient demography, healthcare seeking and care pathways, cancer care and follow-up is obtained from each patient presenting to the NCISL. Prospective data capture was initiated in 2018 and we retrospectively collected data on all new cases diagnosed in 2016 and 2017. Given the retrospective data collection in 2016 and 2017, information on some of the patients who warranted hospital admission and died thereafter were not accessible at time of data entry. This was estimated to be under 300 patients as per NCISL records. The majority of these patients likely presented with advanced disease.

### Study covariates

Cancer stage at diagnosis was defined according to the Tumour, Node, and Metastasis (TNM) staging system, and registered according to the pathological stage, except for neoadjuvant treatments and metastatic disease which were defined by the clinical stage [12]. Invasive tumour grade was defined according to the Elston and Ellis modified Scarff-Bloom-Richardson breast cancer grading system [13]. Oestrogen (ER) and progesterone (PR) receptor status was determined based on the results of immunohistochemistry tests and classified as positive or negative, with a positivity cut-off of 1%. HER-2 status was based on Fluorescent In-Situ Hybridization (FISH) test or when this was not available, on immunohistochemistry [14]. Breast cancer tumor subtype was analysed for patients who had information on ER/PR/HER2 tumour receptor status. Tumor subtypes were defined as luminal A (ER+ and/or PR+ and HER2-), luminal B (ER+ and/or PR+ and HER2+ or - with a high Ki-67), HER2-expressing (ER-, PR- and HER2+) and triple negative (ER-, PR-, and HER2-). Comorbidity was measured using the Charlson Comorbidity Index based on documented comorbidities at diagnosis [15]. We analysed the concordance between guideline recommendations for use of adjuvant therapy for non-metastatic breast cancer and actual rates of delivery of such adjuvant therapies for patients. Guidelines were considered from the National Institute for Health and Care Excellence (NICE) of the United Kingdom and National Comprehensive Cancer Network (NCCN) from the USA [16, 17], which are considered standard in Sri Lanka for adjuvant therapy decision making. Only the absolute indications for adjuvant therapy were included for calculations.

### Statistical analysis

Categorical measures were summarized as numbers with percentages and continuous variables were summarized as means with standard deviation. Chi squared (*χ*^2^) test for trend was used to test for univariate differences in cancer biological characteristics and treatment among groups of women of interest. Statistical analyses were performed in SPSS (Version 24).

### Ethics statement

Ethical approval for this study was obtained from the Faculty of Medicine, University of Colombo Ethics Review Committee (Ref. No. EC-17-068). All methods were performed in accordance with the appropriate ethical guidelines and regulations. Informed consent was obtained from all participants.

## Results

This study included 5181 women with newly diagnosed breast cancer during the 5-year period from 01/01/2016 to 31/12/2020. Distribution of patient and tumour characteristics of the study population is shown in Table 1. The mean age of the women was 56 years [standard deviation (SD) 12, range 21–93 years]. The highest proportion of breast cancer was diagnosed in the 50–59 age group (28%). BMI data were available for 2944 (57%) of patients. Of these 48% (*n* = 1424) were overweight (34%) or obese (14%). The majority were post-menopausal (67%). A total of 1387 (27%) had co-morbidities included in the Charlson score. Charlson scores of 1 and > 1 were observed in 1212 (23%) and 175 patients (4%), respectively. The commonest co-morbidities observed were hypertension (*n* = 1566, 30%), diabetes mellitus (*n* = 1196, 23%), ischaemic heart disease (*n* = 152, 3%) and chronic obstructive pulmonary disease (*n* = 108, 2%).

**Table 1:** Demographic and tumour characteristics of women with breast cancer diagnosed during 2016–2020 at the National Cancer Institute, Sri Lanka

Characteristic	Number of patients (%)
**Age category**	
< 40	429 (9)
40–49	1252 (24)
50–59	1464 (28)
60–69	1359 (26)
70+	677 (13)
**Year of diagnosis**	
2016	973 (19)
2017	1110 (21)
2018	1198 (23)
2019	1064 (21)
2020	836 (16)
**T stage**	
T1	1029 (20)
T2	2909 (56)
T3	619 (12)
T4	619 (12)
Missing	5 (< 1)
**N stage**	
N0	2456 (48)
N1	1700 (33)
N2	693 (13)
N3	327 (6)
Missing	5 (< 0.1)
**M stage**	
M0	4915 (95)
M1	266 (5)
**Stage category**^**a**^	
I	682 (13)
II	2746 (53)
III	1487 (29)
IV	266 (5)
**BMI**	
< 18.5	124 (2)
18.5–24.9	1396 (27)
25–29.9	1009 (20)
> 30	415 (8)
Missing	2237 (43)
**Charlson score**	
0	3794 (73)
1	1212 (23)
> 1	175 (4)
**Histology type**	
Ductal CA	4436 (86)
Lobular CA	222 (4)
Mucinous CA	102 (2)
Papillary CA	56 (1)
Metaplastic CA	30 (1)
Other	87 (2)
Missing	248 (5)
**Grade**	
I	710 (14)
II	2316 (45)
III	1586 (31)
Missing	569 (11)
**LVI**	
Positive	1189 (23)
Negative	3554 (69)
Missing	438 (8)
**ER/PR status**	
Positive	3180 (61)
Negative	1230 (24)
Missing	771 (15)
**HER-2 status**	
Positive	947 (19)
Negative	3129 (60)
Equivocal	302 (6)
Missing	803 (15)
**Subtype**	
Luminal A	2362 (46)
Luminal B	555 (11)
HER-2 enriched	392 (8)
Triple negative	841 (16)
Missing	1041 (20)
**Menopausal Status**	
Peri	364 (7)
Pre	884 (17)
Post	3450 (67)
Unknown	483 (9)

*n* = 5181 unless otherwise specified

^a^Stage category defined by pathological staging except for neoadjuvant treatments and metastatic disease which were defined by the clinical stage

*LVI* Lymphovascular invasion

Approximately two thirds (66%) of the cancers were of early stage (stage I & II) at diagnosis (Table 1). Proportion of early breast cancer by age category ranged from 65% in ≥70-year age category to 66% (in 50–59-year age category).

Approximately 90% of the malignancies were ductal carcinomas which were followed by lobular (4%) and mucinous (2%). Approximately half of the malignancies were grade II tumours (50%), while only 15% were grade I. ER/PR positivity rate was 72% and HER-2 positivity rate was 22%. Of the patients with data available on all biological and receptor characteristics (*n* = 4140, 80%) tumour subtype analysis was done. Of those 57% were luminal A type cancers while the rate of triple negative cancers was 20%.

Treatment characteristics of the women included in the analysis is shown in Table 2. In relation to surgical treatment, 66% women had undergone mastectomy with or without reconstruction as the primary surgical treatment of the breast. When stratified by early (stage I + II) and advanced (III + IV) stage, a larger percentage (73%) of advanced stage patients underwent mastectomy with or without reconstruction than early-stage patients (62%). Of those who had axillary surgery a total of 68% had undergone axillary clearance as the surgical treatment for axillary lymph nodes. Similar to mastectomy numbers, a larger percentage of advanced-stage patients underwent axillary clearance (77%) than early-stage patients (63).

**Table 2:** Treatment characteristics of women with breast cancer diagnosed during 2016–2020 at the National Cancer Institute, Sri Lanka

Type of treatment	Early (%)	Advanced (%)	Total n (%)
**Surgery – Breast**			
Mastectomy only	2101 (61)	1271 (73)	3372 (65)
Mastectomy + reconstruction	32 (1)	9 (1)	41 (1)
Wide local excision	1107 (32)	154 (9)	1261 (24)
No surgery	187 (6)	315 (18)	502 (10)
Missing	1 (< 1)	4 (< 1)	5 (< 1)
**Surgery – Axillary**			
Sentinel LN biopsy	1034 (30)	78 (4)	1112 (22)
Axillary clearance	2174 (63)	1344 (77)	3518 (68)
No axillary surgery	219 (6)	326 (19)	545 (10)
Missing	1 (< 0.1)	5 (< 1)	6 (< 1)
**Neoadjuvant chemotherapy**			
Yes	126 (4)	478 (27)	604 (12)
No	3302 (96)	1275 (73)	4577 (88)
**Adjuvant chemotherapy**			*n* = 4270
Yes	1888 (67)	994 (68)	2882 (68)
No	915 (33)	473 (32)	1388 (32)
**Adjuvant radiotherapy**			*n* = 4270
Yes	1578 (56)	996 (68)	2574 (50)
No	1221 (43)	475 (32)	1696 (33)
**Adjuvant endocrine therapy**			*n* = 4266
Yes	1833 (65)	847 (58)	2680 (52)
No	964 (35)	622 (42)	1586 (31)

Total = 5181, Early (Stage I + II) =3428, and Advanced (Stage III + IV) =1753

Twelve percent of women in our cohort received neoadjuvant chemotherapy (NACT), a larger percentage of which had advanced stage cancers (27%). Neoadjuvant therapy percent included any patient who was started on chemotherapy for non-metastatic cancer with a plan to offer curative surgery.

Adjuvant therapy characteristics were analysed in women (*n* = 4270) who have completed adjuvant therapy or where a decision has already been made against providing adjuvant chemotherapy and radiotherapy (i.e., women who haven’t started or completed their adjuvant therapy were excluded). Of this group, approximately two thirds (68%, *n* = 2882) received adjuvant chemotherapy, which was similarly distributed between early and advanced stage cancer groups. Adjuvant radiotherapy to the breast, chest wall or nodal basins was received by nearly 60% (*n* = 2574) of this subgroup.

Receipt of radiotherapy by different types of surgery to the breast was analysed for women who were diagnosed during 2016–2019 as some patients diagnosed in 2020 are still undergoing chemotherapy and are yet to receive radiotherapy. Of the 4345 eligible patients, 3007 (74%) and 1046 (26%) had undergone mastectomy and wide local excision respectively. Of the mastectomy patients, 1906 (63%) had received radiotherapy while 800 (77%) from the wide local excision group received radiotherapy.

Concordance between guideline recommendations for use of adjuvant therapy for non-metastatic breast cancer and actual rates of delivery of such adjuvant therapies for patients is noted in Table 3. Rate of chemotherapy delivery (neoadjuvant or adjuvant) was 91% for women with node positive breast cancer. Of eligible women 77% (i.e., following breast conserving therapy or with 3+ positive lymph nodes) received adjuvant radiotherapy. Endocrine therapy has been initiated in 88% of women with hormone receptor positive breast cancer while rate of trastuzumab use was 59% among women with HER2 positive breast cancer.

**Table 3:** Concordance between adjuvant treatments delivered to women with non-metastatic breast cancer diagnosed during 2016–2020 at the National Cancer Institute, Sri Lanka versus guideline^a^ recommendations

Indicator	Groups	n (%)
Use of chemotherapy (CT)	CT indicated	2720 (100)
*• CT should be delivered to patients with node-positive disease*	CT delivered	2464 (91)
Use of adjuvant radiotherapy (RT)	RT indicated	2078 (100)
*• RT should be delivered to patients after breast conserving surgery and those with 3+ positive LNs*	RT delivered	1599 (77)
Use of adjuvant endocrine therapy (ET)	ET indicated	3189 (100)
*• Adjuvant ET should be given to all patients with ER and/or PR positive breast positive breast cancer*	ET started	2795 (88)
Use of adjuvant trastuzumab	Trastuzumab indicated	947 (100)
*• Adjuvant trastuzumab should be given to all patients with HER2 positive breast cancer*	Trastuzumab delivered	559 (59)

^a^National Institute of Health and Care Excellence (NICE) and National Comprehensive Cancer Network (NCCN)

## Discussion

In this study, we have described disease characteristics and treatment patterns in a large cohort of women with newly diagnosed breast cancer in Sri Lanka. To our knowledge, this is the largest published cohort of breast cancer patients in Sri Lanka and the most comprehensive especially in relation to treatment characteristics. Based on data from the National Cancer Registry, our cohort includes approximately 40% of all patients in the country [18].

The majority of our patients were post-menopausal at diagnosis. This finding is compatible with a previous study based on national cancer data which has shown a rapid increase in post-menopausal breast cancer in Sri Lanka during 2001–2010 [1]. Furthermore, the mean age at diagnosis was 51 and 52 years, respectively in two previous published studies with data collected between 1994 and 2006 [2, 8] compared to a mean age of 56 years in our study indicating an older age at diagnosis. Sri Lanka has a rapidly ageing population, the fastest in South Asia [19]. A rapidly ageing population combined with rising breast cancer among older women will likely increase breast cancer incidence exponentially among these women who are more likely to have co-morbidities and a poorer survival from breast cancer [20]. For instance, in this study approximately a third of women had hypertension and a quarter had diabetes at the time of breast cancer diagnosis. Increasing age at diagnosis and increasing prevalence of cardiovascular disease and diabetes in Sri Lanka will likely further increase the proportion of women with co-morbidities. This will likely impact the delivery of optimal therapy for breast cancer, which may adversely influence cancer care.

Approximately two thirds of the cancers in this study were of early stage (stage I & II) at diagnosis The proportion of early stage breast cancer in our study is comparable to the two previous studies Balawardana et al. [2] and Mudduwa et al. [9] who have observed 68 and 67% early breast cancers, respectively. Although there are no comprehensive population-based data on cancer stage at diagnosis for comparison, the high rate of advanced breast cancer, which has essentially remained unchanged over last 25 years, is concerning. This reflects the failure of the health system of the country to implement effective strategies aimed at early diagnoses of breast cancer. Our findings of 34% of patients diagnosed with advanced breast cancer (Stage III or IV) is comparable to observations made in other neighboring countries, where in Kerala, India 37% of patients presented with advanced breast cancer [21]. These rates of advanced breast cancer are higher than the rates reported from the greater Southeast Asia region (approximately 20%), but are much lower than the rates reported from African region (approximately 70–75%) [22, 23]. However, the proportion of advanced breast cancer is reported in this study is substantially greater than reported from developed countries where only 15–20% are advanced at diagnosis [24, 25]. Stage at diagnosis is the most important prognostic factor determining survival and greater efforts are needed to promote early diagnosis of breast cancer in the future.

Our study demonstrates a substantial increase in the rate of breast conservation surgery (24%) as compared with the study by Balawardena et al., where the proportion of breast conservative surgery was only 3% despite including similar proportions of stage I and II cancers [2]. This likely reflects better access to mammography, surgeon expertise, as well as greater awareness among women with breast cancer. For instance, the government has expanded mammography services by placing them in every teaching and provincial hospital, which probably has been a major contributor towards increasing rates of breast conserving surgery.

We identified considerable lapses in the concordance between guideline recommendations and the delivery of breast cancer care [16, 17]. For instance, only 77% women with absolute indications for radiotherapy received adjuvant radiotherapy. Furthermore, trastuzumab use was seen in only 59% of women with HER2 positive breast cancer. These observations were comparable to other regions in South Asia. In a smaller cohort of patients in Kerala India, 84% of eligible patients received adjuvant radiotherapy while only 40% of those eligible received trastuzumab [26]. Although the analysis for the possible reasons for the lapses observed were not analysed in this study, it is evident that the provision of breast cancer care has potential for improvement. Access, patient co-morbidities, socioeconomic factors and poor health literacy are some of the known factors that limit use of optimum adjuvant therapy. Difficulties in access for some forms of adjuvant therapy exist in Sri Lanka. For instance, radiotherapy facilities especially linear accelerator facilities are only available at four public health care hospitals in Sri Lanka. NCISL had only one functioning linear accelerator during the study period which probably would have contributed to delays and the limited use of radiotherapy [3].

On the other hand, chemotherapy and endocrine therapy use was found to be quite satisfactory with concordance rates of 90 and 89%, respectively. Although delays in treatments, rates of completion and adherence were not analysed in this study, such high initiation rates indicate a good adherence to guidelines among physicians for therapies which are readily available. The study from Kerala, India has reported substantially lower rates of chemotherapy concordance at 69% for a similar cohort of patients [26]. It appears that regional cancer centres face similar challenges with provision of cancer care, and there may be potential for knowledge transfer and training between similar regional cancer centres which probably would help better understand and learn from experiences of each other to improve quality of care.

The government of Sri Lanka has initiated several strategies with the aim of improving access to treatments and the quality of care for patients with cancer in the country. For instance, a program has been implemented to procure linear accelerators and to station radiation oncology centres in each of the nine provinces of the country to improve the access to radiation therapy [2]. However further action is needed to improve the availability of resources (chemotherapy, endocrine and targeted therapy) to ensure easier access to these services with minimal delays. There is also a need increase the number of surgeons, pathologists, radiologists, oncologists and ancillary staff available to deal with the rapidly rising incidence of breast cancer in Sri Lanka [1, 3].

There are several limitations to our study. While substantial effort was made for complete data capture and validation, there still remains incomplete and missing data mainly in relation to adjuvant therapy and treatment modalities. Logistically, there may be an underrepresentation of late stage breast cancers in 2016 and 2017 given that the charts of some of these patients with advanced disease who warranted hospital admission and died were not accessible at time of data collection. This was estimated to be under 300 patients in our cohort of over 5000 patients (less than 6%). Our cohort only included patients from NCISL. Although patients to NCISL come from all regions of the country, they may not be generalizable to patients treated elsewhere. We may have missed some treatments given at other centres, although efforts were made to capture them through patient interviews. Therefore utilization rates may be slightly under-estimated. Reasons for non-treatment have not been explored in this analysis. Ongoing follow-up is needed to investigate survival outcomes. Furthermore, the as the patients were captured from a tertiary care cancer centre visit, patient and tumour characteristics may be different from the general population because of the higher proportion of more advanced stage cancers seen. Therefore the findings of this study may not be a completely represent the status of breast cancer in Sri Lanka. However, to our knowledge, this is the largest and most comprehensive cohort of patients with breast cancer reported from Sri Lanka.

## Conclusion

The high percentage of advanced breast cancer at diagnosis and high prevalence of comorbidities will make breast cancer care in Sri Lanka challenging. Given that stage at diagnosis is the most important prognostic factor determining survival, greater efforts are needed to promote early diagnosis of breast cancer. Considerable lapses in the concordance between guideline recommendations and the delivery of cancer care warrants closer assessment and intervention.

## Data Availability

The datasets analysed during the current study are not publicly available as the ethics approval for this research does not provide data sharing as the data contain individual patient identification information. However, the data are available from the corresponding author on reasonable request.
